# Anthocyanins in Black Soybean Coats Promote Apoptosis in Hepatocellular Carcinoma Cells by Regulating the JAK2/STAT3 Pathway

**DOI:** 10.3390/ijms26031070

**Published:** 2025-01-26

**Authors:** Yuying Li, Miaomiao Wang, Jinjing Bai, Xin Li, Sheng Xiao, Li Song

**Affiliations:** 1 Key Laboratory of Chemical Biology and Molecular Engineering of Education Ministry, Shanxi Key Laboratory of Biotechnology, Institute of Biotechnology, Shanxi University, Taiyuan 030006, China; 2 Department of Pathology, Brigham and Women’s Hospital, Harvard Medical School, Boston, MA 02115, USA

**Keywords:** black soybean, anthocyanins, hepatocellular carcinoma, apoptosis, network pharmacology, JAK2/STAT3

## Abstract

The use of black soybean (*Glycine max* L.), an edible crop prevalent in Asia, has attracted attention for its hepatoprotective properties. Notably, the anthocyanin components in black soybean coats have shown potential in inhibiting tumor growth. Here, anthocyanins were extracted from black soybean coats using both microwave and water bath methods. The physicochemical characteristics of black soybean coat anthocyanins (BSCAs) and their biological activities were examined. The results from the MTT and EDU assays demonstrated a dose-dependent inhibitory effect of BSCAs on hepatocellular carcinoma HepG2 cells, while leaving normal cells unaffected. Flow cytometry and mitochondrial membrane potential assays revealed that BSCA treatment induces apoptosis in HepG2 cells. A network pharmacology approach was employed to explore the relationship between hepatocellular carcinoma and the active ingredients of BSCAs, identifying the JAK/STAT signaling pathway as a potential target. Molecular docking studies confirmed the interaction between BSCA components and JAK2/STAT3 targets. Subsequent Western blot and qPCR experiments validated that BSCAs promote apoptosis in HepG2 cells by modulating the JAK2/STAT3 signaling pathway.

## 1. Introduction

Hepatocellular carcinoma (HCC) is a prevalent solid malignancy worldwide. Projections indicate a 55.0% annual increase in new HCC cases from 2020 to 2040, leading to rising incidence and mortality rates. By 2040, an estimated 1.4 million diagnoses and 1.3 million deaths are expected, representing a 56.4% increase from 2020 [1]. Treatment options for HCC are limited; surgical resection is effective for early-stage cases or smaller tumors, but most diagnoses occur at advanced stages due to HCC’s initially asymptomatic nature. Liver transplantation, prioritized for advanced cases, faces long waiting times due to a mismatch between organ supply and demand. The management of advanced HCC typically involves treatments such as hepatic arterial chemoembolization (TACE) and radiofrequency ablation, which focus on local disease control but often yield poorer outcomes for systemic or multiple tumors [2]. Sorafenib is a primary systemic treatment for advanced HCC, improving overall survival and progression-free time, but is effective in only 30% of patients, with resistance often developing within six months. Other agents, such as lenvatinib and bevacizumab, face similar resistance issues. Current HCC therapies are challenged by liver toxicity, relapse, and drug resistance, driving research into alternative strategies, including novel drugs targeting specific pathways and drug combinations [3].

Epidemiological studies suggest that a high-fiber, low-fat diet rich in fruits and vegetables may help mitigate the cancer risk [4]. Moreover, natural products have shown promise in reversing multidrug resistance by targeting specific cellular mechanisms, reducing toxicity and adverse effects, and enhancing the effectiveness of anticancer drugs [5,6]. Consequently, these natural products and their bioactive components are considered promising candidates for liver cancer prevention and treatment. Natural polyphenols are known for their ability to safely modulate physiological functions and are commonly found in various fruits and vegetables, demonstrating anticancer properties in different cancer cell types [7,8]. Anthocyanins, a subgroup of flavonoids, are water-soluble pigments responsible for the vibrant colors in plants. They play crucial roles in antioxidant activity, anti-aging processes, immunomodulation, neuroprotection, and antitumor effects [9,10]. Studies have shown that anthocyanins and their glycosides can inhibit tumor cell proliferation while minimally affecting normal cells [11,12]. Extracts rich in anthocyanins from sources such as grapes, red rice germ, and bran have demonstrated inhibitory effects on hepatocellular carcinoma cell growth [13,14]. Additionally, anthocyanins from blueberries exhibit anti-invasive properties against human hepatocellular carcinoma cells, while those from mulberries have been shown to extend survival in rats with HCC [15,16].

Legumes, particularly black beans, are widely consumed around the world, serving as a dietary staple in Central America and being used for culinary and medicinal purposes in Asia. Black soybeans have traditionally been utilized in Oriental medicine for detoxification and anti-inflammatory purposes. They are rich in nutrients such as proteins, essential amino acids, fats, dietary fiber, vitamins, minerals, and polyphenolic compounds, namely phenolic acids, anthocyanins, and isoflavones [17,18]. Among various dark-hued foods, black soybean coats have a higher concentration of anthocyanins, which exhibit superior antioxidant and hypoglycemic properties [19]. Recent research indicates that extracts from black soybean coats can modulate gut microbiota and serum metabolites, improving conditions such as type 2 diabetes. Additionally, the phenolic content of hull extracts shows significant antioxidant and anti-inflammatory effects [20]. Since 1997, the World Cancer Research Fund/American Institute for Cancer Research has recognized the anticancer potential of legumes [21]. Studies by Wei et al. demonstrated that anthocyanins from black bean coats, especially the key component cyanidin-3-O-glucoside(C3G), inhibit tumor progression and enhance survival rates in a dose-dependent manner [22]. Furthermore, Dong et al. isolated bioactive compounds from black soybean skins that inhibit the growth of Caco-2 human colon cancer cells, HepG2 human hepatocellular carcinoma cells, and MCF-7 human breast cancer cells [23]. Notably, phenolic compounds extracted from black lentils were found to be more effective in inducing apoptosis and suppressing the proliferation of colon cancer cells compared to other anthocyanin-rich extracts from sources like sorghum and red grapes [24]. Yamamoto et al. conducted a study showing that black bean skin extract effectively mitigated non-alcoholic fatty liver disease and non-alcoholic hepatitis [25]. The research highlighted that cyanidin-3-O-glucoside, a prominent compound of anthocyanins commonly found in black beans and black rice, can reverse oxaliplatin resistance and enhance its therapeutic effect on hepatocellular carcinoma [26]. Consequently, we sought to investigate whether anthocyanins directly impact hepatocellular carcinoma cells and elucidate their mechanisms of action, which we explore in this study.

The JAK/STAT signaling pathway is a critical pathway involved in cellular processes such as growth, differentiation, maturation, apoptosis, and immune regulation [27]. The JAK2/STAT3 pathway is commonly upregulated in various human tumors. Current research indicates that the JAK2/STAT3 pathway plays a crucial role in promoting cancer cell growth, invasion, metastasis, and anti-apoptosis, particularly in triple-negative breast cancer, melanoma, and other tumor diseases [28,29,30]. Studies have identified STAT3 as a key regulatory gene within the JAK2/STAT3 pathway, with elevated STAT3 levels observed in different cancer types [31]. For instance, Yang et al. showed that matrine induces mitochondrial apoptosis in cholangiocarcinoma cells by inhibiting the JAK2/STAT3 pathway [32]. The discovery highlights the significance of the JAK2/STAT3 signaling pathway in modulating apoptosis to inhibit the formation of tumors.

This study aimed to extract BSCAs using microwave and water bath methods for a more efficient extraction process. Network pharmacology, integrating systems biology, computational biology, and network biology, provides a comprehensive framework for understanding the mechanisms of various components and their effects on multiple targets. This interdisciplinary approach has significantly advanced modern drug development and clinical applications. We intend to elucidate the mechanism of action of BSCAs on liver cancer cells through cellular experiments and network pharmacology analysis. These efforts are undertaken to offer insights into the development of black bean-derived therapeutics, in alignment with the concept of medicine and food being homologous.

## 2. Results

### 2.1. Optimal Conditions for BSCA Extraction Using Water Bath

**Figure 1:**
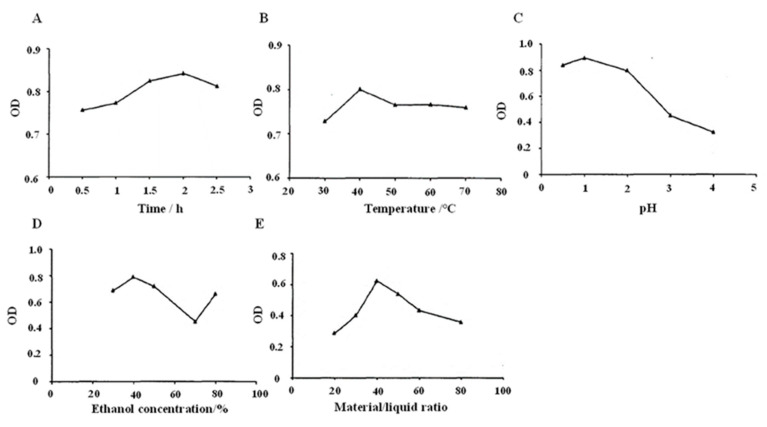
Optimal conditions for BSCA water bath extraction. (**A**) Effects of different extraction times (0.5, 1, 1.5, 2, and 2.5 h) on water extraction of BSCAs. (**B**) Effect of different extraction temperatures (30, 40, 50, 60, and 70 °C) on BSCA extraction from water. (**C**) Effect of different extraction pH levels (0.5, 1.0, 2.0, 3.0, and 4.0) on water extraction of BSCAs. (**D**) Effects of different extracted ethanol concentrations (30%, 40%, 50%, 60%, 70%, and 90%) on BSCA water extraction. (**E**) Effect of different extraction solid–liquid ratios (1:20, 1:30, 1:40, 1:50, and 1:60) on BSCA water extraction.

The extraction of anthocyanins increased with incubation time, peaking at 2 h, after which, their levels began to decline (Figure 1A). Notably, the anthocyanins demonstrated greater solubility in the extraction medium than in the solvent. Furthermore, as the temperature rose, the absorbance of the anthocyanins also increased, before eventually decreasing. This temperature increase facilitated enhanced anthocyanin solubility in the extraction medium and boosted diffusion coefficients (Figure 1B). The stability of the anthocyanins was significantly affected by the ambient pH, with the optimal stability observed at an acidic pH of 1.0 (Figure 1C). The absorbance levels of the anthocyanins initially increased with the rising ethanol concentrations but subsequently decreased (Figure 1D). This trend reflects the varying solubilization effects of anthocyanins at different ethanol concentrations. The optimal ethanol concentration for anthocyanin extraction was found to be 40%, as lower concentrations improved solubilization, while higher concentrations reduced precipitation efficiency (Figure 1E).

### 2.2. Optimal Conditions for BSCA Extraction Using Microwave

**Figure 2:**
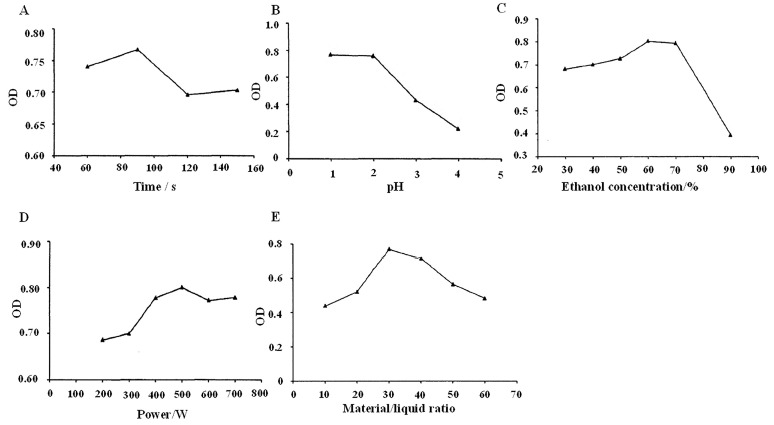
Optimal conditions for BSCA microwave extraction. (**A**) Effect of extraction time (60, 90, 120, and 150 s) on microwave extraction of BSCAs. (**B**) Effect of pH level (1.0, 2.0, 3.0, and 4.0) on microwave extraction of BSCAs. (**C**) Effect of ethanol concentration (30%, 40%, 50%, 60%, 70%, and 90%) on microwave extraction of BSCAs. (**D**) Effect of microwave power (200, 300, 400, 500, 600, and 700 W) on BSCA extraction. (**E**) Effect of solid–liquid ratios (1:10, 1:20, 1:30, 1:40, 1:50, and 1:60) on microwave extraction of BSCAs.

The most effective extraction time with the microwave technique was 90 s (Figure 2A). The optimal extraction results were achieved at pH levels of 1.0 and 2.0 (Figure 2B). The ideal ethanol concentration for microwave extraction was 60% (Figure 2C). As the microwave power increased, absorbance gradually rose, peaking at 500 W (Figure 2D). The optimal material-to-liquid ratio was established at 1:30 (Figure 2E). Compared to the water bath method, the microwave-assisted technique demonstrated greater efficiency (Table 1 and Table 2), supporting its use for anthocyanin extraction in this study.

### 2.3. Physicochemical Properties and Composition of BSCAs

**Figure 3:**
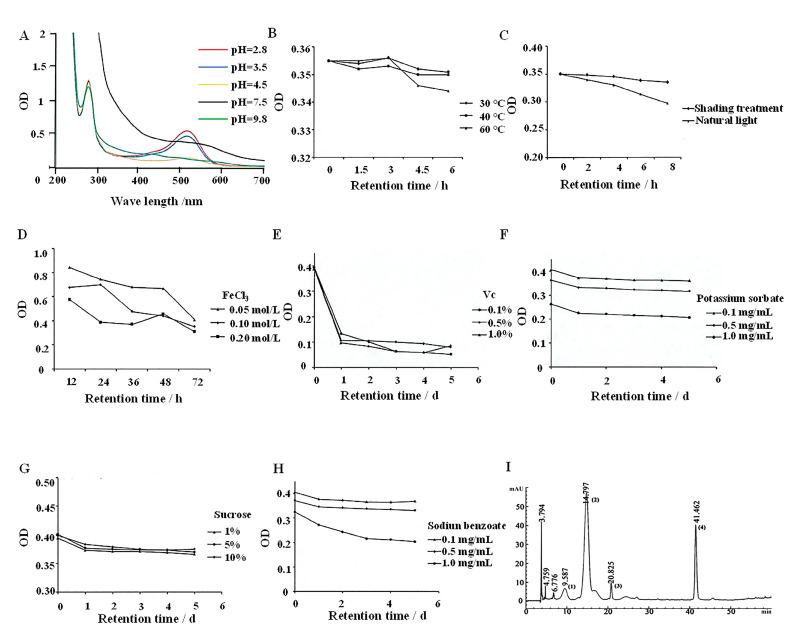
Physicochemical properties and composition of BSCAs. (**A**) Anthocyanin spectra at different pH values (2.8, 3.5, 4.5, 7.5, and 9.8). (**B**) Effect of temperature (30, 40, and 60 °C) on anthocyanin stability. (**C**) Effect of light exposure on anthocyanin stability. (**D**) Effect of Fe^3+^ (0.05, 0.1, and 0.2 mol/L) on the stability of anthocyanins. (**E**) Effect of Vc (0.1%, 0.5%, and 1%) on anthocyanin stability. (**F**) Effect of potassium sorbate (0.1, 0.5, and 1.0 mg/mL) on the stability of anthocyanins. (**G**) Effect of sucrose (1%, 5%, and 10%) on anthocyanin stability. (**H**) Effect of sodiun benzoate (0.1, 0.5, and 1.0 mg/mL) on anthocyanin stability. (**I**) HPLC for the detection of BSCA content. Compared with the standard sample, the components of the BSCAs are (1) delphinidin-3-glucoside (D3G), (2) cyanidin-3-O-glucoside (C3G), (3) petunidin 3-glucoside (Pt3G), and (4) cyanidin (Cy).

The spectrograms of anthocyanins dissolved in solutions with varying pH levels (Figure 3A) reveal distinct absorption peaks at approximately 280 nm and 520 nm. Notably, at a pH of 3.5 or lower, a prominent absorption peak appears at 513 nm. As the pH reaches 4.5, this visible absorption peak diminishes and disappears entirely under alkaline conditions. Heating results in a gradual decline in anthocyanin absorbance, with a more pronounced decrease at higher temperatures, indicating overall good thermal stability at body temperature (Figure 3B). Under natural light, absorbance decreases, while only minimal changes are observed under light-protected conditions (Figure 3C). The effects of Na^+^ and K^+^ on anthocyanin stability are negligible (Figure S1A,D), whereas Ca^2+^ shows some influence (Figure S1B) and Mg^2+^ provides a mild color-protecting effect (Figure S1C). In contrast, Fe^3+^ significantly impacts stability (Figure 3D), and redox agents severely destabilize the anthocyanins (Figure 3E and Figure S1F). Preservatives such as potassium sorbate and sodium benzoate exhibit concentration-dependent effects on anthocyanin stability, with higher concentrations leading to decreased stability (Figure 3F,H). Sugars, however, have a minimal impact on anthocyanin stability (Figure 3G and Figure S1E).

The composition and purity of the BSCAs were analyzed via HPLC, revealing the presence of delphinidin-3-glucoside (D3G, 6.7%), cyanidin-3-O-glucoside (C3G, 64%), petunidin-3-glucoside (Pt3G, 3.4%), and cyanidin (Cy, 20.5%) (Figure 3I).

### 2.4. Antitumor Effects of BSCAs

**Figure 4:**
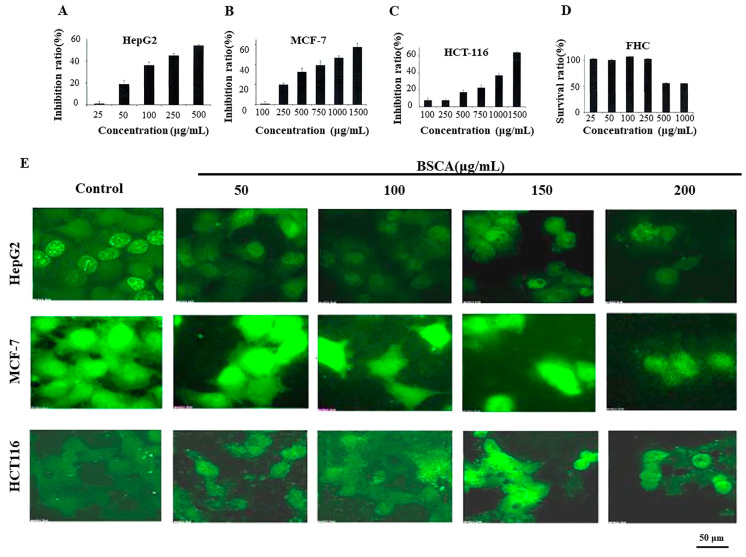
Screening of BSCA inhibition effect on tumors. (**A**–**C**) Inhibition of BSCAs (25, 50, 100, 250, and 500 μg/mL) on HepG2 cells for 48 h. BSCAs (100, 250, 500, 750, 1000, and 1500 μg/mL) inhibit MCF-7 cells and HCT-116 cells for 48 h. (**D**) BSCAs (25, 50, 100, 250, 500, and 1000 μg/mL) on FHC cells viability at 48 h (**E**) BSCAs (50, 100, 150, and 200 μg/mL) on HepG2, MCF-7, and HCT-116 cells at 48 h of the EDU assay.

The inhibitory effects of BSCAs on specific tumor cell types were evaluated using MTT assays, which showed a dose-dependent inhibitory effect of anthocyanins on all the cancer cell lines. Specifically, the inhibitory effect on HepG2 liver cancer cells is most pronounced, with an inhibition rate of 50% at a concentration of 250 μg/mL. It is preliminarily judged that BSCAs have a better effect on liver cancer cells. When BSCAs act on normal FHC cells, with increasing concentration, cell death occurs at concentrations above 500 μg/mL due to osmotic pressure. At the concentrations of 250 μg/mL and below, there is no significant impact on the survival of normal cells (Figure 4A–D). Therefore, concentrations equal to or less than 250 μg/mL were considered safe for BSCA treatment, and subsequent experiments utilized these lower concentrations.

Additionally, the EdU assay (Figure 4E) revealed a significant decrease in fluorescence intensity in the HepG2 cells with increasing anthocyanin concentrations, while no significant differences were observed in the MCF–7 and HCT116 cells compared to the control group. Combined with the MTT results, it appears that anthocyanins inhibit the proliferation of HepG2 hepatocellular carcinoma cells more effectively than MCF-7 breast cancer or HCT116 colon cancer cells. Consequently, subsequent investigations primarily focused on hepatocellular carcinoma cells.

### 2.5. BSCAs Promote Apoptosis in Hepatocellular Carcinoma Cells

**Figure 5:**
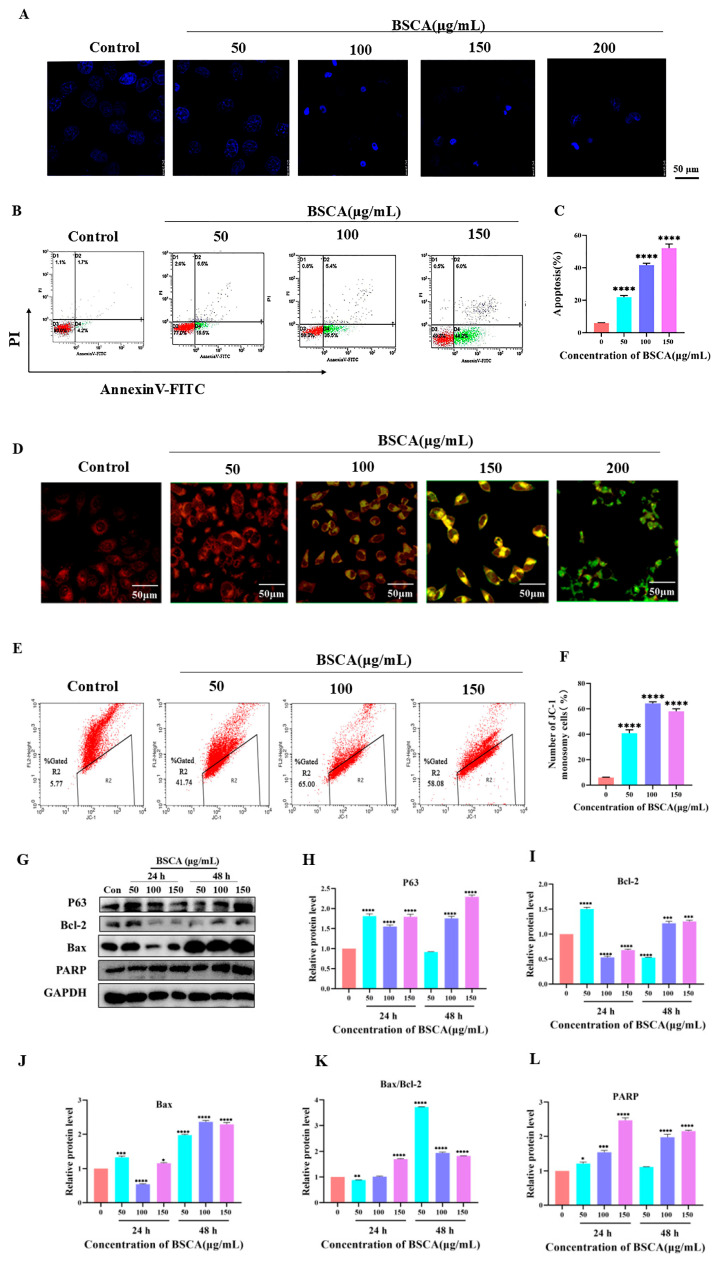
Characterization of BCSA acting on HepG2 cells. (**A**) Effect of BSCAs (50, 100, 150, and 200 μg/mL) on HepG2 nuclei. (**B**,**C**) BSCAs (50, 100, and 150 μg/mL) after 24 h of HepG2 cells, apoptosis, and its analysis. (**D**–**F**) BSCAs (50, 100, 150, and 200 μg/mL) after 24 h of HepG2 cells, confocal imaging, quantification, and analysis of mitochondrial membrane potential. (**G**–**L**) BSCAs (50, 100, and 150 μg/mL) on HepG2 cells for 24 h and 48 h to express apoptosis-related proteins in HepG2 cells. * *p* < 0.05, ** *p* < 0.01, *** *p* < 0.001, **** *p* < 0.0001 compared with the control group.

The BSCA treatment of HepG2 cells led to cell shrinkage and solidification, and nuclear chromatin condensation is the key feature of apoptosis (Figure 5A), and the BSCA-induced apoptosis was dose-dependent (Figure 5B,C). The JC-1 assay showed a transition from red to green fluorescence in the HepG2 cells with higher BSCA concentrations, indicating a progressive decrease in the mitochondrial membrane potential (Figure 5D). The quantification of this reduction, measured by green fluorescence expression using flow cytometry, revealed an increase followed by a decrease in the number of JC-1 monomers as the BSCA concentration increased, compared to the control (Figure 5E,F). Further confirmation of the reduction in the mitochondrial membrane potential was obtained through western blot analysis of Bcl-2 and Bax (Figure 5G), which showed an increase in the Bax/Bcl-2 ratio, (Figure 5I–K). Additionally, there was a notable rise in the expression levels of the pro-apoptotic proteins P63 and PARP (Figure 5H,L).

A parallel investigation was conducted using another hepatocellular carcinoma cell line, SMMC7721, which showed results consistent with those observed in the HepG2 cells (Figure S2A–H).

### 2.6. Network Pharmacology Analysis and Validation of BSCAs’ Action in Hepatocellular Carcinoma

**Figure 6:**
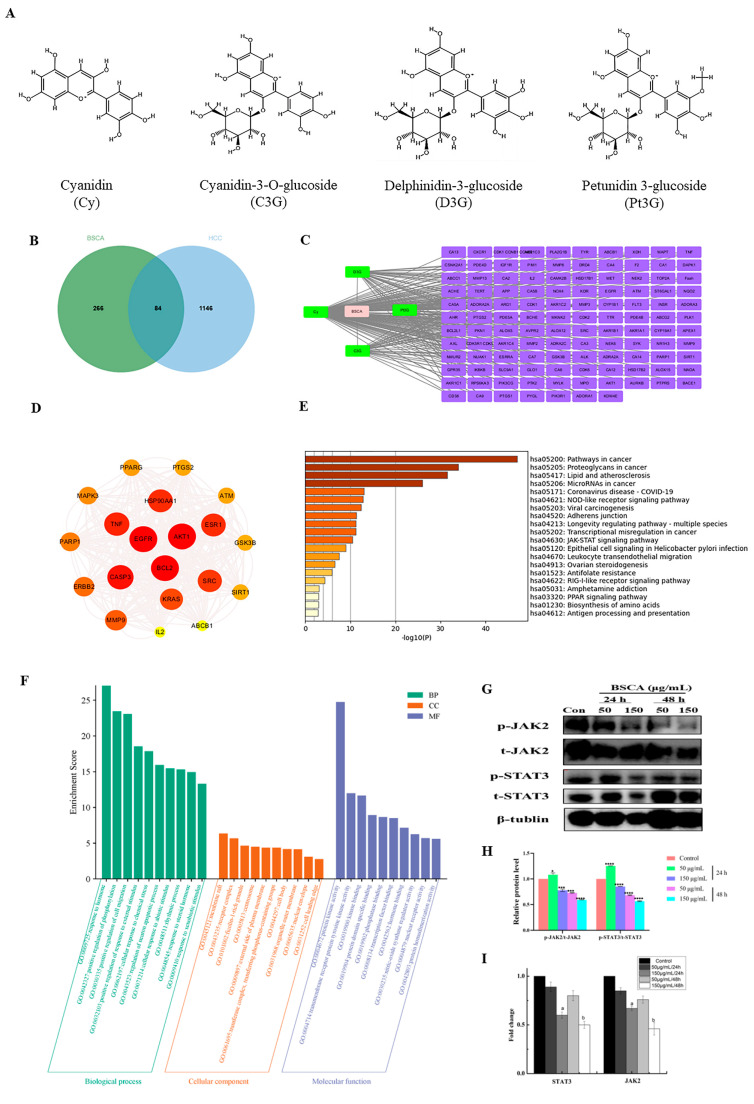
A bioinformatics analysis and the validation of BSCAs after the application of hepatocellular carcinoma cells. (**A**) The structural formula of the four principal components of BSCAs. (**B**) The intersection targets of BSCAs and HCC. (**C**) Targets of BSCAs. (**D**) High-density interaction network of BSCAs intersection targets. (**E**) KEGG enrichment analysis of intersecting targets. (**F**) GO enrichment analysis of intersecting targets. (**G**,**H**) The effect of BSCAs on the expression of the key proteins of the JAK/STAT signaling pathway. (**I**) Effect of BSCAs on *JAK2* and *STAT3* gene expression. * *p* < 0.05, *** *p* < 0.001, **** *p* < 0.0001 compared with the control group. Different letters indicate significant difference (*p* < 0.05).

In order to elucidate the molecular mechanisms and targets of BSCAs in HCC, we conducted a network pharmacological screening to predict the specific components of BSCAs relevant to this disease. The analysis revealed 84 intersections between the targets of BSCA components and liver cancer (Figure 6B). A subsequent visualization and high-density protein network screening identified key apoptotic proteins, including Bcl-2 and CASP3, among the high-density action proteins.

The enrichment analysis of the intersecting targets indicated involvement in processes such as hormone response, cell phosphorylation regulation, apoptosis, and membrane composition, with a predominant molecular function related to protein kinase activity. A further KEGG enrichment analysis highlighted the involvement of these intersecting proteins in cancer signaling pathways (Figure 6C–E). Considering the role of BSCAs in promoting apoptosis and the kinase activity of the key intersecting proteins identified in the GO enrichment analysis, our investigation focused on the JAK/STAT signaling pathway. This pathway was selected due to its mechanism involving phosphorylation cascades and its role in modulating apoptosis.

To validate these findings, we conducted a Western blot analysis to examine the levels of p-JAK2/t-JAK2 and p-STAT3/t-STAT3 proteins—pivotal components of the signaling pathway—in hepatocellular carcinoma cells (Figure 6G,H and Figure S2I,J). The results indicated that the BSCA treatment led to modifications in the expression levels of p-JAK2/t-JAK2 and p-STAT3/t-STAT3. Additionally, the qPCR analysis of the *JAK2* and *STAT3* genes showed a downward trend in their expression levels (Figure 6I and Figure S2K). These findings suggest that BSCAs influence the JAK2/STAT3 signaling pathway in hepatocellular carcinoma.

### 2.7. Docking of BSCA Components with JAK and STAT Protein Molecules

**Figure 7:**
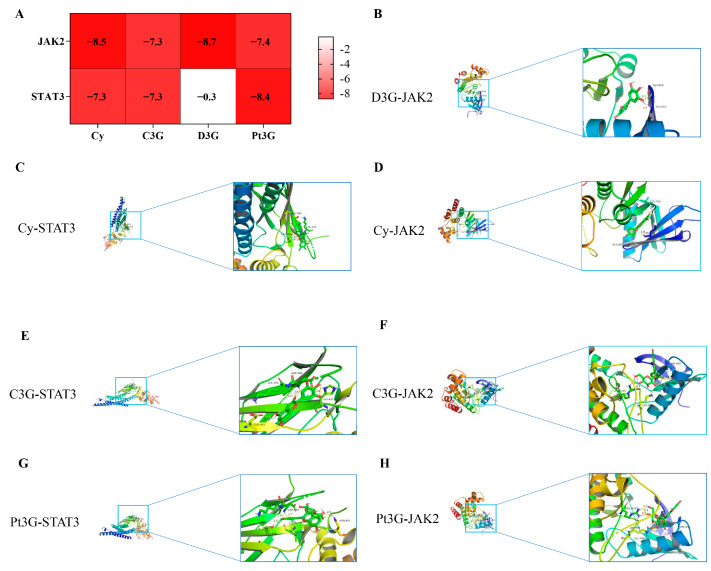
The binding capacity of various components of BSCAs to JAK, STAT proteins. (**A**) Docking score heat map of various components of BSCAs with JAK, STAT proteins. (**B**–**H**) The three cases in which the ingredients scored the highest for protein docking.

Molecular docking is a fundamental theoretical simulation technique used in drug design, aimed at analyzing the interactions between molecules, such as ligands and receptors, to predict their binding modes and strengths. To further explore the specific functions of the key constituents of BSCAs in this context, we conducted a molecular docking analysis involving these constituents and the JAK/STAT protein targets. The docking results revealed that, with the exception of D3G, all the components had docking scores below −7 kJ/mol with the JAK2 and STAT3 proteins, indicating strong binding capabilities (Figure 7A). Notably, D3G and Cy demonstrated binding energies of −8.7 kJ/mol and −8.5 kJ/mol, respectively, with JAK2, while Pt3G exhibited a docking score of −8.4 kJ/mol with STAT3, indicating a superior binding affinity compared to the other components.

## 3. Discussion

Black soybean hulls are rich in diverse bioactive compounds that exhibit significant roles, including antioxidant, anti-inflammatory, and hepatoprotective activities [17,33,34]. Among these, anthocyanins stand out for their health benefits and high market demand. Their antioxidant properties and bioactivities are crucial for developing health-promoting products, such as functional foods, nutritional supplements, and food colorants [35]. Optimal extraction methods are essential for enhancing the utility and market value of anthocyanins. Various techniques, including solvent extraction, microwave-assisted extraction, enzymatic extraction, and ultrasound-assisted extraction, are commonly used for anthocyanin extraction [36,37,38,39]. In this study, we selected water bath and microwave extraction—two cost-effective and widely employed methods. We optimized extraction parameters such as temperature, time, pH, material-to-liquid ratio, and power to enhance anthocyanin extraction efficiency. Comparisons between the two methods revealed that microwave extraction is not only more cost-effective but also yields a higher extraction rate than water bath extraction.

Anthocyanins are highly sensitive to various environmental factors, including light, heat, pH, and oxygen. Their degradation and discoloration can significantly impact the sensory quality of food products and diminish their bioactivity [40]. Our study focused on the physicochemical characteristics of BSCAs, revealing that exposure to light can lead to degradation, resulting in reduced color intensity and bioactivity. Additionally, high temperatures compromise anthocyanins’ stability, making them more susceptible to pyrolysis or denaturation [41]. The solubility of anthocyanins is crucial for their extraction efficiency and application methods, with variations noted under different pH conditions. Interactions with sugars and food additives during processing and storage are inevitable, and high levels of sugar degradation products can threaten anthocyanins’ stability [42]. Complex formation between anthocyanins and metal ions can also induce color changes and reduce their functional properties [43]. Our investigation aimed to assess the potential of anthocyanins from black soybean coats as food colorants, considering the impact of co-additives. The results indicated that, within a specific temperature range, the thermal stability of anthocyanins is satisfactory, although precautions such as protection from light are necessary. While sugars have minimal effects on the stability, redox agents can significantly impair it. Among various metal ions, Na^+^ and K^+^ have minor effects, Mg^2+^ offers slight color protection, Ca^2+^ shows some impact, and Fe^3+^ has a pronounced effect on anthocyanin stability.

Anthocyanins, a subgroup of flavonoids, have been extensively studied for their potential in cancer prevention due to their inhibitory effects on various cancer types [44]. Previous research has primarily focused on their role in preventing colorectal cancer, demonstrating their ability to impede disease progression through multiple mechanisms [45,46]. Studies on hepatocellular carcinoma have shown that anthocyanins extracted from grapes can hinder the proliferation of cancer cells. Anthocyanins from hairy beans and white grapes have demonstrated anti-invasive effects on human hepatocellular carcinoma cells, as indicated in previous studies [47]. Moreover, research has shown that anthocyanins sourced from radish possess the ability to modulate oxidative stress in these cells [48]. These compounds can also disrupt the hepatoprotective effects of the Wnt4/β-linker pathway, potentially extending the survival of rats with hepatocellular carcinoma. Additionally, anthocyanins have been shown to inhibit the epithelial-mesenchymal transition of hepatocellular carcinoma cells triggered by epidermal growth factor [49].

The potential inhibitory effects of BSCAs on tumor cell proliferation were examined through MTT assays. These assays indicated that the BSCAs did not significantly harm the normal FHC cells. Notably, the BSCAs exhibited the most pronounced inhibitory effects on the hepatocellular carcinoma HepG2 cells, as well as the colon cancer HCT-116 and the breast cancer MCF-7 cells. The significant impact of the BSCAs on the HepG2 cells was further confirmed through EDU assays. Consequently, the HepG2 and the SMMC7721 cells were selected for further investigation to validate the anti-hepatocellular carcinoma properties of BSCAs. Our findings revealed that increasing the concentrations of the BSCAs led to morphological changes in the cells, such as rounding, contraction, reduced volume, solidified nuclei, and condensed nuclear chromatin—characteristics indicative of apoptosis [50].

We assessed apoptosis in the hepatocellular carcinoma cells using flow cytometry. Apoptosis can occur through various pathways, including endogenous, exogenous, and endoplasmic reticulum stress pathways. Among these, the endogenous apoptosis pathway, also known as the mitochondrial apoptosis pathway, is primarily regulated by the Bcl-2 family of proteins [51]. Anthocyanidins, recognized for their strong antioxidant properties, have been shown to influence the oxidative state of cells, reduce mitochondrial membrane potential, and induce apoptosis [52]. Based on our observations, we postulated that the induction of apoptosis in hepatocellular carcinoma cells by BSCAs occurs via the mitochondrial apoptotic pathway. Our analysis of mitochondrial membrane potential changes demonstrated a gradual decrease with increasing anthocyanin concentrations, as indicated by the shift from JC-1 aggregates emitting red fluorescence in healthy mitochondria to JC-1 monomers emitting green fluorescence. Furthermore, the BSCAs modulated the expression levels of key proteins: the Bax/Bcl-2 ratio increased, and pro-apoptotic proteins P63 and PARP showed significant elevation. These findings suggest that BSCAs influence the mitochondrial membrane potential, leading to apoptosis in hepatocellular carcinoma cells. To investigate the specific mechanisms and functional components of BSCAs, we conducted bioinformatics predictions by analyzing the intersection of BSCA components and hepatocellular carcinoma targets, along with GO and KEGG enrichment analyses. This analysis identified relevant signaling pathways, particularly the JAK/STAT pathway, which has been implicated in the pathogenesis of various solid tumors, including hepatocellular carcinoma. Therapeutic strategies targeting the JAK/STAT pathway have gained significant attention in health and disease contexts [53]. Notably, the JAK2/STAT3 pathway is crucial for regulating apoptosis, a vital process for promoting cell death [54,55]. Given the observed pro-apoptotic effects of BSCAs in hepatocellular carcinoma cells, we focused on their influence on the JAK/STAT pathway. Subsequent examinations of BSCA-activated hepatocellular carcinoma cells revealed alterations in the gene expression of *JAK2* and *STAT3*, as well as changes in the levels of p-JAK2 and p-STAT3 proteins. Molecular docking simulations of various BSCA components with JAK2 and STAT3 proteins indicated specific components with higher binding affinities, suggesting their potential to induce apoptosis in hepatocellular carcinoma cells by modulating the JAK2/STAT3 pathway. The current therapeutic drugs often face challenges such as liver toxicity, recurrence, drug resistance, and high cost during treatment, for example, immune checkpoint inhibitors like nivolumab, tyrosine kinase inhibitors targeting VEGF/VEGFR like sorafenib, CYP17A1 inhibitor abiraterone, and DNA synthesis inhibitor cisplatin. Compared with existing liver cancer treatment drugs, BSCAs have the advantage of being derived from natural products, with minimal toxicity to normal cells and a low extraction cost, and BSCAs act on JAK2/STAT3 targets to inhibit the proliferation of liver cancer cells, providing a new direction for the development of liver cancer drugs.

## 4. Materials and Methods

### 4.1. Materials and Reagents

Black soybean (*Glycine max* L.) seeds were purchased from the Shanxi Academy of Agricultural Sciences in Taiyuan, China. The cyanidin-3-O-glucoside was standard (Solarbio, Beijing, China); the methanol, formic acid, and acetonitrile were of chromatographic grade (Keomio, Tianjin, China); we used a liquid chromatography column (Venusil XBP-C18, Bonna-Agela, Tianjin, China). All the other reagents were domestic, analytical, pure reagents. The human colon cancer HCT116 cells, human normal intestinal epithelial cells FHC cells, human breast cancer MCF-7 cells, human hepatocellular carcinoma HepG2 cells, and SMMC7721 cells were kept in our laboratory. We used the following: a Mitochondrial Membrane Potential Assay Kit (JC-1) and a Hoechst 33,258 Staining Reagent (Biotronix, Lake Oswego, OR, USA); an Annexin V-FITC Apoptosis Detection Kit (Pharmingen-Becton Dickinson, NJ, USA); and an EDU Cell Proliferation Detection Kit (KGI Bio, Jiangsu, China).

### 4.2. BSCA Extraction by Water Bath

Five aliquots of 0.4 g of black soybean coat powder were weighed and mixed in a 50% ethanol solution under a pH of 2.0 at a ratio of 1:40. Subsequently, they were subjected to extraction periods at 50 °C for varying durations of 0.5–2.5 h, then we isolated the anthocyanin extracts. We diluted them and measured their absorbance values at 530 nm. All the other conditions were constant; the optimal extraction method under each condition was determined by changing the temperature (30, 40, 50, 60, 70 °C), pH levels (0.5, 1, 1.5, 2, 2.5), ethanol concentration (30%, 40%, 50%, 60%, 70%, 80%), and material-liquid ratio (1:20, 1:30, 1:40, 1:50, 1:60).

### 4.3. Microwave-Assisted Extraction of BSCAs

Obtaining the black soybean coat powder involved dissolving four portions of 0.4 g powder in a 50% ethanol solution at pH 2.0, with a material–liquid ratio of 1:40 and the microwave power set at 400 W. The resulting extract was then subjected to centrifugation at 7500 r/min for 5 min following microwave exposure durations of 60 s, 90 s, 120 s, and 150 s. Subsequently, the absorbance value at 530 nm was measured after dilution to ascertain the optimal microwave extraction time. Further investigations included adjusting the extraction power to 200–700 W to determine the ideal power setting; all the other conditions were constant; the optimal extraction method under each condition was determined by changing the pH levels (0.5–4.0), ethanol concentration (30–90%), and material–liquid ratio (1:10, 1:20, 1:30, 1:40, 1:50, 1:60).

### 4.4. Purification of BSCAs

The activated D–101 macroporous resin was applied to the column and equilibrated, followed by the dissolution of crude anthocyanin powder in a reduced amount of double-distilled water. The solution was then subjected to centrifugation to eliminate any residual insoluble particles before being introduced to the chromatographic column for adsorption. Upon reaching the saturation point, the adsorbent resin underwent a washing process with distilled water until the effluent was devoid of any turbidity. Subsequently, the eluate was eluted using 70% ethanol. The resulting eluate was collected, concentrated under reduced pressure at 40 °C, and then subjected to freeze-drying to yield a dark red, purified BSCA powder, preserved in light at −20 °C.

### 4.5. Comparison of Extraction Rate and Yield

The BSCAs were extracted under the optimum extraction conditions of the water bath and microwave-assisted extraction methods. Under the optimal conditions of the extraction, the volume of the first immersion after centrifugation was V1, and the absorbance was measured, A1; after the second immersion after centrifugation, the volume of the first immersion liquid was combined as V2, and the absorbance was measured after combining the extracts as A2. The volume of all the immersion liquids was combined as the total of V after the centrifugation of the last immersion, and the absorbance value was measured as the total of A. The calculation of the method: the quantitative black soybean coats were combined with the extracting agent several times, until the extract was nearly colorless; then, we recorded the total volume (Va) and the total absorbance (Aa) and calculated the extraction rate of each time according to the extraction rate = A × V/(Aa × Va) × 100%. The BSCAs were extracted under the optimum conditions: rotary evaporation followed by vacuum freeze-drying to obtain anthocyanin powder. The mass of the powder was weighed, m. The calculations were made on the basis of the rate of obtaining = m/M × 100%, where M is the mass of the raw material used, and m is the mass of the anthocyanin extracted to the anthocyanin.

### 4.6. Identification of BSCAs

The BSCAs extracted from the black soybean coats underwent purification using D101 macroporous resin. Subsequently, a portion of the sample was formulated into a specific concentration solution for the analysis of purity and content through Agilent high-performance liquid chromatography (HPLC), performed online. The HPLC method employed the following online conditions: mobile phase A, consisting of a 0.3% formic acid solution of chromatographic grade; mobile phase B, comprising 100% acetonitrile of chromatographic grade, a Venusil XBP C18(L) LC column temperature set at 25 °C, a flow rate of 0.8 mL/min, and a detection wavelength of 513 nm. An injection volume of 10 μL was utilized, with elution gradients described by the formula A90% B10% for 0−5 min, A85% B15% for 5–45 min, A75% B25% for 5–45 min, A75% B25% for 5–45 min, and, finally, A75% B25% for 45–60 min.

### 4.7. Physical and Chemical Properties of Anthocyanins

#### 4.7.1. Absorption Spectra of BSCAs

The anthocyanins were obtained through an optimized extraction process, followed by rotary evaporation and freeze-drying at −40 °C to yield the crude anthocyanin powder, which was stored at −20 °C in darkness. A 1 g portion of the crude anthocyanin extract powder was dissolved in distilled water to a volume of 100 mL. The resulting solution was divided into 5 aliquots and the pH levels were adjusted to 2.8, 3.5, 4.5, 7.5, and 9.8 using hydrochloric acid. Subsequently, UV-visible spectrophotometry was employed to analyze and determine the optimal pH for the anthocyanins.

#### 4.7.2. Stability of BSCAs

A specific quantity of anthocyanin powder was dissolved in double-distilled water and the pH was regulated to 3.5 using dilute hydrochloric acid. We dissolved a certain amount of anthocyanin powder in double distilled water and adjusted the pH to 3.5 with dilute hydrochloric acid. The conditions of the temperature, light, metal ions, preservatives, sugar, redox agents, and other conditions were changed, respectively, to study the stability of the BSCAs under different conditions.

### 4.8. Cell Culture and Cell Viability Assay

The cells were cultured in an RPMI-1640 medium supplemented with 10% fetal bovine serum and 1% penicillin–streptomycin and maintained at 37 °C with 5% CO_2_. The impact of the BSCAs on the cell proliferation was assessed using the MTT assay. Specifically, the cells were seeded at a density of 5 × 10^3^ cells/mL in a 100 μL complete RPMI-1640 medium in 96-well microtiter plates. Following adhesion in a fresh medium, the cells were exposed to varying concentrations of BSCAs (50, 100, 150, 200, 250, 500 μg/mL) for 48 h. The control groups were treated with equivalent volumes of the cell culture medium. Subsequently, 20 μL of the MTT solution (5 mg/mL) was added to each well and incubated at 37 °C for 4 h. Upon removal of the medium, 150 μL of DMSO was added to dissolve the formazan crystals by agitation in a shaker for 5 min at room temperature. The absorbance at 490 nm was then measured using an enzyme-linked immunosorbent assay reader. The percentage of the inhibition was determined using the formula: the percentage of inhibition = (1 − mean experimental absorbance/mean control absorbance) × 100%.

### 4.9. EDU Cell Proliferation Assay

The cells were seeded at a density of 3 × 10^5^ cells/well in 6-well microtiter plates and treated with varying concentrations of BSCAs for 48 h post-adhesion. The control group received an equivalent volume of the cell culture medium. Subsequently, 50 μM EDU (5-ethynyl-2′-deoxyuridine) was introduced into 6-well culture plates, and the cells were incubated for an additional 2 h. Subsequently, 1 mL of 4% paraformaldehyde solution was applied to each well for fixation at 25 °C. After discarding the fixative, the wells were rinsed twice with 3% bovine serum albumin (BSA) and phosphate-buffered saline (PBS), followed by the addition of 1 mL of 0.5% Triton X-100 to each well. The plates were then left at 25 °C for 20 min. After two additional washes with 3% BSA, 500 µL of Click-iT EDU solution was added to each well of the 6-well plates and incubated for 30 min at 25 °C in darkness. After staining with Hoechst 33342 dye for 20 min, coverslips were mounted onto glass slides with anti-fluorescence quencher, air-dried, and sealed with nail polish. The samples were visualized using a delta-vision microscope.

### 4.10. Morphological Changes of the Cell Nucleus

A small drop of the cell culture solution was placed in the middle of the bottom of each well of a 6-well cell plate so that the coverslip clamped into it was fixed to the bottom. Each well was inoculated with 1 × 10^5^ cells in the logarithmic growth phase. After overnight wall attachment, different concentrations of the BSCA solution were added, and the untreated group was supplemented with an equal volume of the medium, with 2 replicate wells in each group. After 48 h of drug action, we carefully removed the cell culture stock solution, washed with PBS 2–3 times with gentle shaking, added 500 µL of Hoechst’s staining solution of 5 µg/mL to each well, and incubated them for 30 min at 25 °C in a dark environment; then, we washed them with PBS 2–3 times, each time for 5–10 min; next, we took out the coverslips and inverted them onto the slides with a little anti-fluorescence quencher. Then, we marked the slides and sealed them with nail polish after a little drying. Lastly, we placed them under a fluorescence microscope to observe the nuclear morphology of the cells.

### 4.11. Apoptosis Assay

When the cells grew to the logarithmic growth phase, cell passaging was performed; the cells were inoculated into 6-well plates at 1 × 10^6^ per well, cultured for 24 h to wait for the cells to attach to the wall, and then BSCAs were added to treat the cells for 24 h. The medium was collected, and the cells were washed in the wells with PBS 2–3 times, digested by adding trypsin (without EDTA), and centrifuged to collect the cells. Then, we added PBS to adjust the number of cells to (1 × 10^6^ cells)/mL, resuspended the cells with 300 µL 1×Binding Buffer, and added 3 µL PI and 9 µL Annexin V–FITC; next, we blew slowly and then reacted the coloration for 30 min at room temperature in the dark, then we went on the machine and detected the apoptosis of the cells by flow cytometry.

### 4.12. Measurement of Mitochondrial Transmembrane Potential (MMP, Δψm)

A small drop of the cell culture solution was placed in the middle of the bottom of each well of a 6-well cell plate, then each well was inoculated with 1 × 10^5^ cells in logarithmic growth phase and incubated at 37 °C in 5% CO_2_ incubator until the growth was about 70% of the plate area and the cells were treated with different concentrations of BSCAs for 24 h. Two parallel controls were set for each group. When the treatment was completed, the cells were processed according to the JC-1 detection kit and observed by laser confocal microscope. The quantitative experiments were consistent with the observational experiments. After the treatment, the cells were treated according to the instructions of the JC-1 kit for collecting the cells, and then flow cytometry was used to detect the number of JC-1 monomers at the wavelength of 514 nm and to analyze the changes in the mitochondrial membrane potential.

### 4.13. Western Blot Assay

The cells were treated with BSCAs for 24 h or 48 h. The cells were collected, washed twice with cold PBS, and then ice-lyzed in a cold RIPA extraction buffer for 30 min to prepare the cellular protein extracts. The supernatant was centrifuged at 12,000 r/min for 15 min in a high-speed freezer centrifuge. The protein concentrations were assayed with the BCA Protein Assay Kit. Then we added a Loading Buffer to the EP tube, boiled it for 5 min, centrifuged it for 3 min at 8000 r/min, and then performed SDS-PAGE electrophoresis with 30 μg of protein per well; after electrophoresis, we transferred the protein to the PVDF membrane by the wet transfer method for 1–2 h. The PVDF membrane was immersed in 5% skimmed milk powder for 1.5 h, and the primary antibody was incubated at 4 °C overnight. The PVDF membrane was immersed in 5% skimmed milk powder for 1.5 h. The primary antibody was incubated at 4 °C overnight and washed with TBST 3 times for 10 min each time, and the secondary antibody was incubated at room temperature for 2 h. The membrane was immersed in chemiluminescent solution (liquid A: liquid B = 1:1), and then the exposure was observed by chromatography in the dark room after color development for 2–3 min.

### 4.14. qPCR Assay

Following the manufacturer’s directions, total RNA was extracted from the cells using Trizol. Subsequently, the RNA was reverse transcribed to cDNA using a Prime Script RT Master Mix. The SYBR green master mix was then used for quantitative real-time PCR amplification and detection according to the manufacturer’s protocol. The primers were shown as follows (Table 3). PCR reaction as follows: 94 °C for 5 min, followed by 40 cycles of 94 °C for 30 s, 55 °C for 30 s, and 72 °C for 30 s. *GAPDH* was employed as a reference gene, and the relative expression levels were analyzed using the double-strand DNA method.

### 4.15. Network Pharmacology

The structural formulas of the anthocyanin components Cy, C3G, D3G, and Pt3G were downloaded from the PubChem database (https://pubchem.ncbi.nlm.nih.gov). In order to get more accurate predictions, we obtained the drug targets in the ChEMBL (https://www.ebi.ac.uk/chembl, accessed on 25 September 2024) and Swiss Target Prediction database (https://www.expasy.org/resources/swissdrugdesign, accessed on 25 September 2024) to get the drug action targets, which were merged and de-weighted to get the drug targets in GeneCards database (https://www.genecards.org). “Liver cancer” was entered as the keyword, and the disease targets were obtained after screening. The drug targets and disease targets were mapped in cytoscape 3.6.0 software and the high-density interacting proteins were selected. The drug targets and disease targets were selected as intersecting targets on the website of Microbiology (https://www.bioinformatics.com.cn) and visualized for mapping. The intersecting targets were entered into the Metascape database (https://metascape.org), and the GO and KEGG enrichment analysis results of the intersecting targets were obtained. The Microbiology website was used for the visualization and mapping to obtain the GO enrichment analysis results.

### 4.16. Molecular Docking

The 3D structures of the BSCA components were downloaded from the PubChem database (https://pubchem.ncbi.nlm.nih.gov/), and the 3D structures of the JAK (7F7W) and STAT3 (6njs) proteins were downloaded from the PDB database (https://www.rcsb.org) and then optimized using the PyMOL2.4.0 software to dehydrogenate them for optimization, target docking them with each component of the BSCAs using AutoDock Vina molecular docking tool.

### 4.17. Statistical Analysis

The data obtained from all the experiments were expressed in the form of the mean ± standard deviation (X ± S); the number of experimental repetitions was *n* ≥ 3. In the data analysis of this study, we first employed a one-way ANOVA to examine the significant differences between the multiple groups. If the data did not meet the assumptions of normality or the homogeneity of variance, the Kruskal–Wallis H test was used as a non-parametric alternative to ensure the reliability of the results. Additionally, when comparing only two extraction methods, we used a *t*-test to assess whether the mean difference between them was statistically significant. The analysis of the data was carried out by SPSS 13.0 software, with ap < 0.05 or bp < 0.01 representing statistically significant differences. The graphs were made with the Origin 2018 software, GraphPad Prism 8.

## 5. Conclusions

In conclusion, our study suggests that BSCAs can induce apoptosis in hepatocellular carcinoma cells and modulate the JAK2/STAT3 signaling pathway in hepatocellular carcinoma cells.

## Figures and Tables

**Table 1:** The comparison of anthocyanin extraction rate.

Method	Extraction Times	Volume (mL)	A_530_	Extraction Efficiency
Microwave extraction	1	13	0.695	72.9%
	2	27	0.414	90.2%
Water extraction	1	14	0.482	68.2%
	2	30	0.280	84.9%

**Table 2:** The comparison of anthocyanin yield.

Method	Raw Material Quality (g)	Anthocyanin Quality (g)	Yield
Microwave extraction	0.8	0.1073	13.4%
Water extraction	0.8	0.0876	10.9%

**Table 3:** Primer sequences.

Genes	Primers
*Jak 2*	Forward:5′-TCTGGGGAGTATGTTGCAGAA-3′
*STAT3*	Forward: 5′-CAGCAGCTTGACACACGGTA-3′
*GAPDH*	Forward:5′-CCCATGTTTGTTGTTGGTGTC-3′

## Data Availability

The original contributions presented in the study are included in the article/Supplementary Material; further inquiries can be directed to the corresponding author.

## Supplementary Materials

The supporting information can be downloaded at https://www.mdpi.com/article/10.3390/ijms26031070/s1.
