# Supplementary material for: Anthocyanins in Black Soybean Coats Promote Apoptosis in Hepatocellular Carcinoma Cells by Regulating the JAK2/STAT3 Pathway
Source: Int J Mol Sci. 2025 Jan 26;26(3):1070. doi: 10.3390/ijms26031070 (PMC11817063; doi:10.3390/ijms26031070)
Supplement: Supplementary file 1 [file ijms-26-01070-s001.zip › Figure S1.(1).pdf]

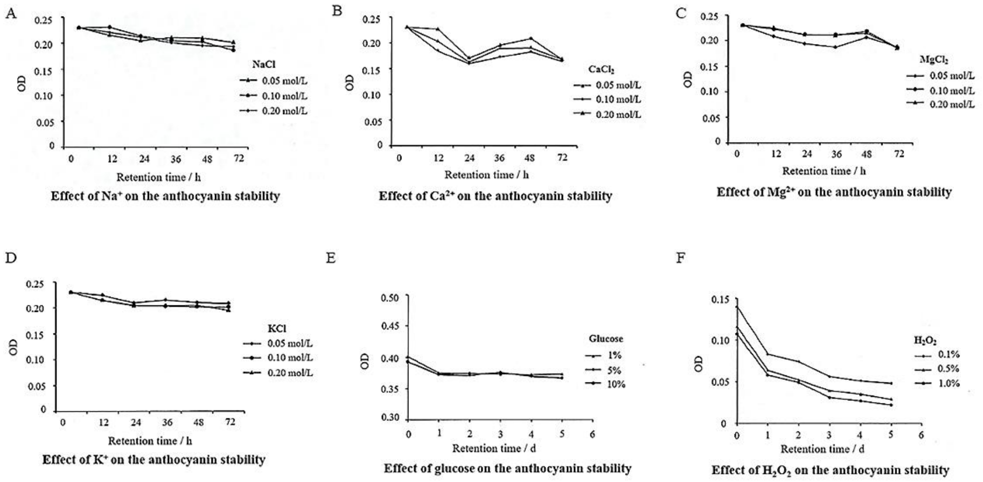

Figure S1. Physicochemical properties and composition of BSCA. (A) Effect of Na^+ (0.05, 0.1, 0.2 mol/L) on the stability of anthocyanins. (B) Effect of Ca^{2+} (0.05, 0.1, 0.2 mol/L) on the stability of anthocyanins. (C) Effect of Mg^+ (0.05, 0.1, 0.2 mol/L) on the stability of anthocyanins. (D) Effect of K^+ (0.05, 0.1, 0.2 mol/L) on the stability of anthocyanins. (E) Effect of Glurose (1%, 5%, 10% mg/mL) on anthocyanin stability. (F) Effect of H_2O_2 (0.1%, 0.5%, 1%) on anthocyanin stability.
