# Supplementary material for: Anthocyanins in Black Soybean Coats Promote Apoptosis in Hepatocellular Carcinoma Cells by Regulating the JAK2/STAT3 Pathway
Source: Int J Mol Sci. 2025 Jan 26;26(3):1070. doi: 10.3390/ijms26031070 (PMC11817063; doi:10.3390/ijms26031070)
Supplement: Supplementary file 1 [file ijms-26-01070-s001.zip › Figure S2(1).pdf]

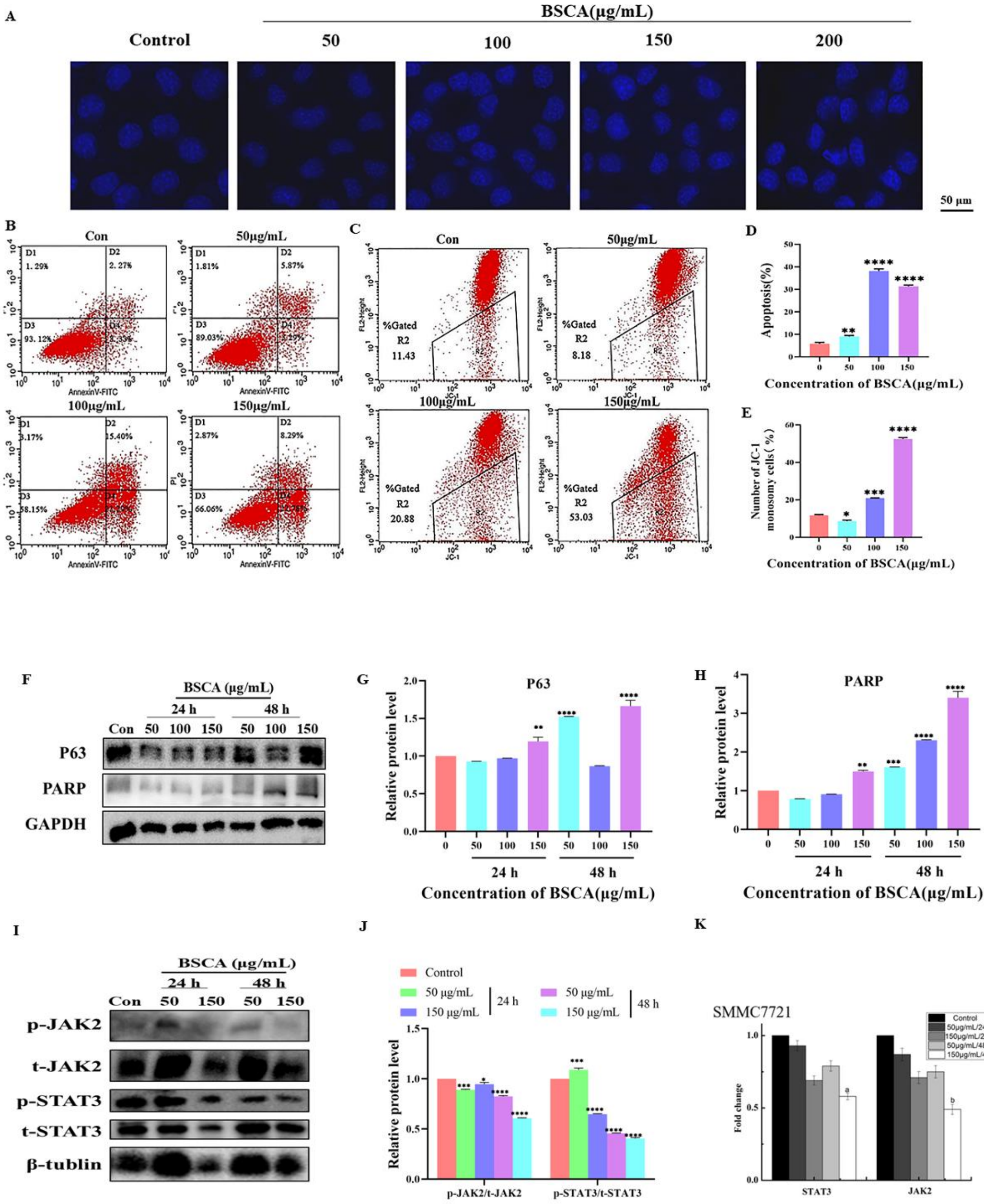

Figure S2. Characterization of BSCA acting on SMMC7721 cells. (A) Effect of BSCA (50, 100, 150, 200 μg/mL) on SMMC7721 nuclei. (B-E) BSCA (50,100,150 μg/mL) after 24 h of SMMC7721 cells, apoptosis and its analysis, quantification, and analysis of mitochondrial membrane potential. (F-H) BSCA (50,100,150 μg/mL) on SMMC7721 cells for 24 h and 48 h to express apoptosis-related proteins in SMMC7721 cells. (I, J) Effect of BSCA (50, 150 μg/mL) on the expression of p-JAK and p-STAT proteins. (K) Effect of BSCA on *JAK* and *STAT* genes expression.
